# Profiling the MAPK/ERK dependent and independent activity regulated transcriptional programs in the murine hippocampus *in vivo*

**DOI:** 10.1038/srep45101

**Published:** 2017-03-28

**Authors:** Nils Blüthgen, Mirjam van Bentum, Barbara Merz, Dietmar Kuhl, Guido Hermey

**Affiliations:** 1 Institute for Theoretical Biology and Institute of Pathology, Charité - Universitätsmedizin Berlin, 10117, Berlin, Germany; 2 Institute for Molecular and Cellular Cognition, Center for Molecular Neurobiology Hamburg, University Medical Center Hamburg-Eppendorf, 20251, Hamburg, Germany

## Abstract

Activity-dependent alteration of the transcriptional program is central for shaping neuronal connectivity. Constitutively expressed transcription factors orchestrate the initial response to neuronal stimulation and serve as substrates for second messenger-regulated kinase signalling cascades. The mitogen-activated protein kinase ERK conveys signalling from the synapse to the nucleus but its genetic signature following neuronal activity has not been revealed. The goal of the present study was to identify ERK dependent and independent activity regulated transcriptional programs in the murine hippocampus. We used generalized seizures combined with the pharmacological intervention of MEK activation as an *in vivo* model to determine the complete transcriptional program initiated by ERK after neuronal activity. Our survey demonstrates that the induction of a large number of activity-regulated genes, including Arc/Arg3.1, Arl5b, Gadd45b, Homer1, Inhba and Zwint, is indeed dependent on ERK phosphorylation. In contrast, expression of a small group of genes, including Npas4, Arl4d, Errfi1, and Rgs2, is only partially dependent or completely independent (Ppp1r15a) of this signalling pathway. Among the identified transcripts are long non-coding (lnc) RNAs and induction of LincPint and splice variants of NEAT1 are ERK dependent. Our survey provides a comprehensive analysis of the transcriptomic response conveyed by ERK signalling in the hippocampus.

Activity dependent alteration of the transcriptional program of neurons is a key mechanism for shaping neuronal connectivity in the brain. Such neuronal plasticity contributes to a variety of physiological and pathological processes in the developing and adult brain. These include learning and memory, response to injury, ischemia and drugs, epileptogenesis and neurodegenerative and neuropsychiatric disorders^1^,^2^. Signalling from the synapse to the nucleus induces gene expression and provides a mechanism for translating synaptic activity into persistent changes^3^,^4^. Numerous studies identified genes whose expression is altered by different neuronal plasticity inducing stimuli^5^, but there is limited information on the transcriptional programs initiated by particular signal transducing pathways.

Constitutively expressed transcription factors are thought to orchestrate the initial transcriptional response to neuronal stimulation^5^. These transcription factors frequently serve as substrates for second messenger-regulated kinase signalling cascades. Among them is the well described and highly conserved mitogen-activated protein kinase (MAPK) pathway^6^,^7^. The extracellular regulated kinase (ERK) is a prototype of MAPK and the activating pathway is characterized by a core of three kinases. The first is a MAP kinase kinase kinase (Raf-1 or B-raf), which phosphorylates the second MAP kinase kinase (MEK). MEK finally activates the two ERK isoforms ERK1 and ERK2 by phosphorylation. This cascade mediates the transmission of signals from the synapse to cytoplasmic and nuclear effectors^8^,^9^,^10^. Phosphorylated ERKs can activate transcription factors directly, such as ELK-1 that translocates after activation from the cytoplasm to the nucleus of neurons^11^, or indirectly via intermediary kinases, such as CREB^6^,^12^. Activation of the ERK pathway is critical for neuronal plasticity related events and initial work demonstrated an absolute requirement for ERK activity in the induction of long-term potentiation (LTP) in hippocampal slices^13^ and that ERK activity regulates gene transcription and LTP *in vivo*^14^. Subsequently it was shown that ERK activation is important for LTD and plays a critical role in mammalian learning and memory^15^,^16^,^17^,^18^,^19^,^20^,^21^,^22^. Additional studies suggest that ERK phosphorylation is required for proper development of neuronal functions and its inhibition leads to the development of autistic phenotypes in mice^23^.

However, the transcriptional program in neurons initiated by ERK has only been partially elucidated. Most studies used a candidate gene approach rather than performing a genome wide analysis. For example the ERK/MAPK dependent transcription of the activity-regulated gene Arc/Agr3.1 has been well documented^24^,^25^,^26^, but it is still not clear whether the interplay between ERK signalling and other transduction pathways during plasticity induces all activity-regulated genes or if there is a group of genes whose induction by activity is independent of ERK activation. Our objective was to determine the transcriptional program initiated by ERK after neuronal activity in the murine hippocampus. To this end, we used chemically provoked seizures to induce strong, synchronized neuronal activity in combination with the MEK inhibitor SL327 and performed a genome wide analysis of the initiated transcriptional program.

## Results

### Kainic acid induced seizures result in transient phosphorylation of ERK in the hippocampus which can be blocked by a small molecule MEK inhibitor

**Figure 1:**
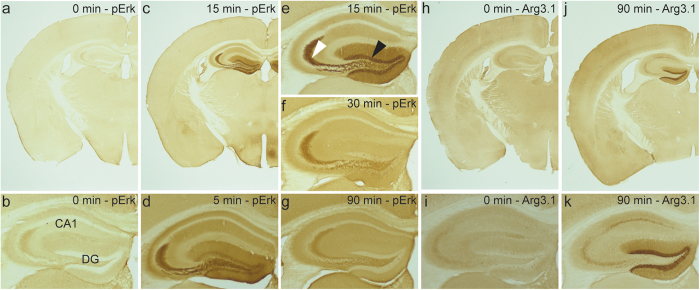
Seizures trigger ERK phosphorylation. (**a–k**), Seizures were induced in mice by intraperitoneal injection of kainic acid and animals sacrificed at indicated time points after seizure onset. Parallel coronal brain sections were immunostained for p-ERK (**a–g**) or for Arc/Arg3.1 (**h–k**). CA1, field CA1 of the hippocampus; DG, dentate gyrus. White arrowhead in e indicates the mossy fiber zone, black arrowhead in e points at the granular cell layer of the dentate gyrus.

We triggered seizures in mice to induce strong synchronous neuronal activity in the hippocampus. To assess the time course of ERK activation after kainic acid provoked seizures we analysed brain sections of mice sacrificed 5, 15, 30, and 90 minutes after seizure onset. The phosphorylation and activation of ERK was monitored by using phospho-site-specific antibodies. Immunohistological analysis revealed that phosphorylation of ERK occurs within minutes after seizure onset in the hippocampus (Fig. 1a–g). Most intense staining was observed after 5 to 15 minutes and the p-ERK signal was strongly reduced after 90 minutes. The granular cell layer and molecular layer of the dentate gyrus were predominantly stained (Fig. 1e). In addition, the mossy fiber zone exhibited strong labelling. We observed an increased p-ERK staining in the dendritic layers but not in the cell bodies of pyramidal neurons in CA1-CA3. The observed pattern is in agreement with previous results obtained after generalized seizures in mice^24^,^27^. We immunostained parallel sections of the brains dissected at the different time points after seizure onset for Arc/Arg3.1. The activity-induced protein was detectable 90 minutes after seizure onset in the granular cell layer and molecular layer of the dentate gyrus and in the pyramidal cells in CA1-3 (Fig. 1h–k). At this time point the p-ERK signal was already reduced. Thus, our observations are in accordance with the concept that ERK phosphorylation precedes activity dependent gene expression.

**Figure 2:**
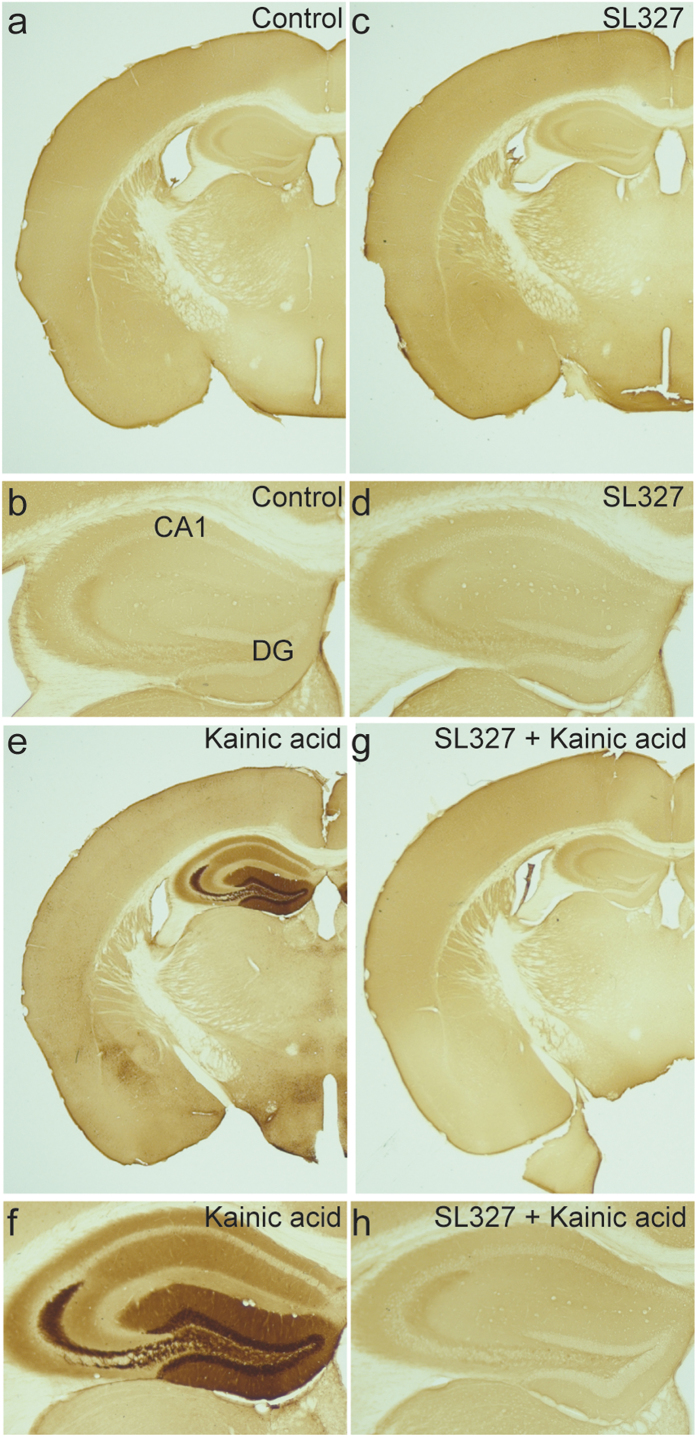
The MEK inhibitor SL327 blocks seizure induced ERK phosphorylation in the dentate gyrus. (**a–h**) Coronal mouse brain sections immunostained for p-ERK. (**a**,**b**) vehicle treated control; (**c**,**d**) brain section of a mouse treated with SL327 for 90 minutes; (**e**,**f**) brain section of a mouse sacrificed 15 minutes after onset of seizures; (**g**,**h**) brain section of a mouse treated with SL327 60 minutes before intraperitoneal kainic acid injections and sacrificed 15 minutes after onset of seizures. Note the complete inhibition of hippocampal ERK phosphorylation (**g**,**h**). CA1, field CA1 of the hippocampus; DG, dentate gyrus.

Next we analysed the *in vivo* inhibition of ERK phosphorylation by intraperitoneal injection of the blood-brain barrier-penetrating MEK inhibitor SL327 which has no significant effect on a variety of other kinases such as CAMKII, PKC or PKA^15^,^28^ (Supplemental Fig. 1). The inhibitor was applied one hour before kainic acid injections and the animals developed strong seizures. Application of the inhibitor resulted in a complete blockade of ERK phosphorylation in the hippocampus (Fig. 2a–h).

### Transcriptome analysis identifies MEK/ERK dependent genes

**Figure 3:**
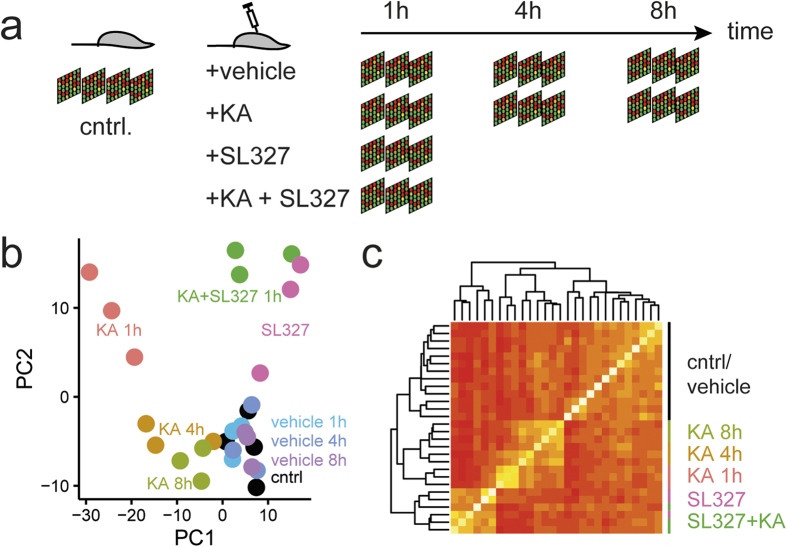
Experimental design and principal component analysis. (**a**) Schematic outline of the experimental design for the microarray analysis. Samples of four untreated mice were used as baseline controls and per indicated treatment group and time point, samples of 3 mice were used. RNA extracted from each sample was hybridized to one microarray. (**b**) Principal component analysis of the 1000 most varying genes demonstrates similar behaviour of replicates. (**c**) Unsupervised cluster analysis of the correlation between the 1000 most varying transcripts shows that the expression profiles of each experimental group cluster together. Cntrl, control; KA, kainic acid.

Next we treated mice with vehicle, SL327, kainic acid or SL327 combined with kainic acid and obtained hippocampal tissue for microarray analysis from animals sacrificed 1 hour after seizure onset or from time matched controls. In addition, we included in our analysis untreated control animals; mice treated with kainic acid sacrificed 4 and 8 hours after onset of seizures and respective time matched controls treated with vehicle only (Fig. 3a). RNA extracted from one hippocampus was hybridized to one microarray, and we measured four replicate animals for the untreated controls and three time matched replicates for all other treatments. Using principle component (PC) analysis of the 1000 top varying genes (Fig. 3b), we found that expression data from controls and vehicle treated animals group together. Kainic acid treatment had the strongest global effect on the transcriptome (change in PC1 and also PC2 at t = 1 h), and the transcriptome showed the strongest response 1 h after treatment. Kainic acid treatment with inhibitor or inhibitor alone resulted in hardly any change in the major PC (PC1), but in changes in PC2 similar to kainic acid treatment, suggesting that PC2 contains genes that are regulated whenever MAPK signalling is modulated, regardless of inhibition or activation. When using unsupervised clustering on the correlation of transcriptomes (Fig. 3c), we found that again controls and vehicle treatment form one cluster, kainic acid treatment forms a second cluster, and inhibitor treatment forms a distinct cluster. Taken together, these analyses confirm that expression profiles of parallel treated animals were mostly similar, and that the data clearly separates effects of kainic acid treatment and pathway inhibition.

**Figure 4:**
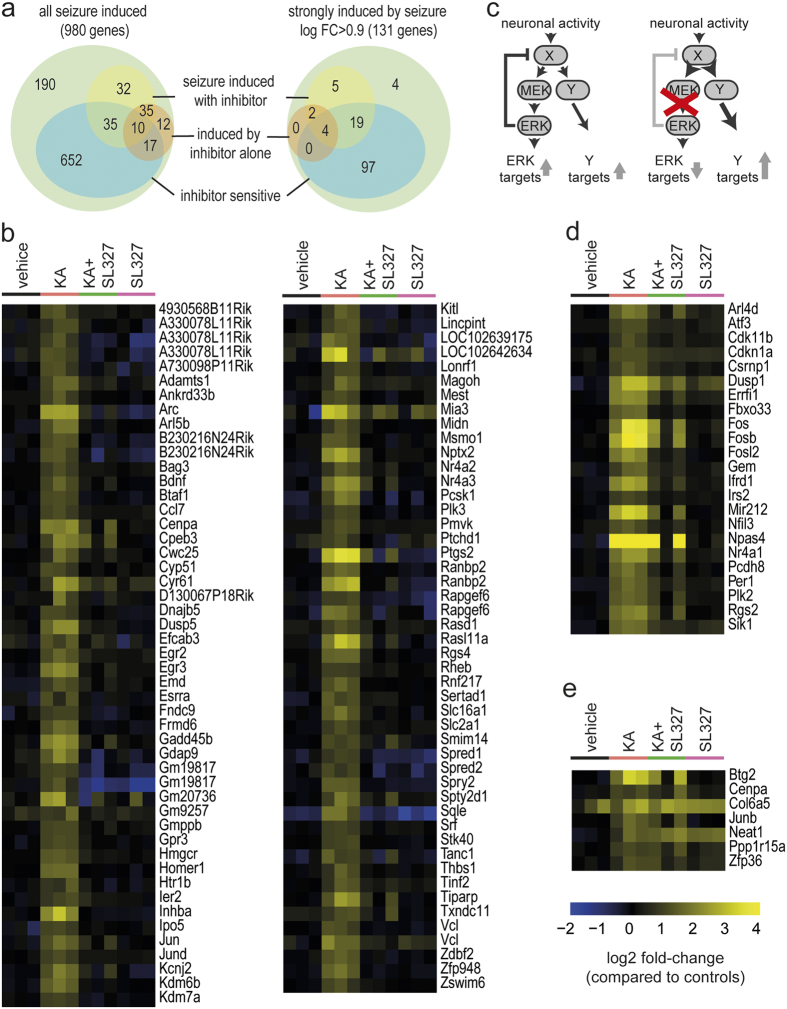
Expression profiles of genes induced by neuronal activity and their dependence on MEK activation. (**a**) Venn diagram indicating the classification of all genes induced by seizure (left) and of those genes that are minimally induced by a log fold change of 0.9 (right). (**b**) Heat map providing an overview of fully MEK dependent gene expression induced by neuronal activity. (**c**) Schematic showing the induction of parallel pathways by MEK inhibition through feedback control. (**d**) Heat map of genes whose expression is partially dependent on MEK activity. (**e**) Heat map of MEK independent gene expression induced by neuronal activity. Yellow indicates upregulation, blue indicates downregulation compared to control animals.

To identify genes robustly induced after seizure we discarded genes with very low expression levels and used a stringent cut-off for differential expression (FDR < 0.05). Following this definition, we identified 980 genes induced by neuronal activity (Fig. 4a). To identify which activity-induced genes are regulated by MEK/ERK signalling, we classified these genes as “inhibitor sensitive” if the presence of the MEK inhibitor significantly attenuated the induction. We called those genes that showed an induction in the presence of the inhibitor “seizure induced with inhibitor” and defined genes that were induced by the inhibitor alone. Intersecting these groups (Fig. 4a left) showed that out of 980 induced genes 190 could not be classified as they were neither significantly induced in the presence of the inhibitor nor significantly affected by the inhibitor. Inspection of these genes revealed a moderate induction of expression and statistical testing between groups was not of sufficient power. We therefore focus on the 131 significantly induced genes with an induction of at least 0.9 log fold change, of which most could be classified according to our criteria (Fig. 4a right).

120 of the top 131 activity-regulated genes were classified as inhibitor sensitive. Out of these, the majority (97 probe sets corresponding to 89 genes) were not significantly induced in the presence of a MEK inhibitor, therefore we termed these fully MEK signalling dependent genes (Fig. 4a,b). The identified transcripts comprise a large number of previously described activity-regulated genes, such as Arg3.1/Arc or Homer1, but also poorly characterized genes, non-coding RNAs and long non-coding RNAs (lncRNAs). Expression of 23 genes was induced after seizures and their induction was only partially reduced in the presence of the MEK inhibitor (Fig. 4d). These activity-regulated genes were classified as “partially MEK signalling dependent” and comprise several previously described activity-regulated genes, such as Npas4, Rgs2, Arl4d and Errfi1. A small group of seven genes was induced after seizures and this induction was not hampered in the presence of the MEK inhibitor (Fig. 4a,e). These activity-regulated genes were classified as “MEK signalling independent”. Within this group of genes a particular induction pattern was observed, e. g. for the lncRNA Neat1. Seizures induce transcript levels of these genes in absence and presence of MEK inhibition and MEK inhibitor treatment alone also resulted in upregulation. Such regulation of expression is expected for genes that are regulated by parallel pathways. MEK/ERK signalling is known to contain strong negative feedbacks. If the MEK/ERK pathway is inhibited, these feedbacks are relieved and can cross-activate parallel pathways^29^, and these in turn can induce genes (Fig. 4c). Two genes in the MEK-independent set (Neat1, Col6a5) and four of the partially MEK dependent genes (Dusp1, Atf3, Cdkn1a and Sik1) showed this regulation albeit with variable strength (Fig. 4a,d,e).

### *In-situ* hybridization validates classes of MEK/ERK dependent and independent genes

**Figure 5:**
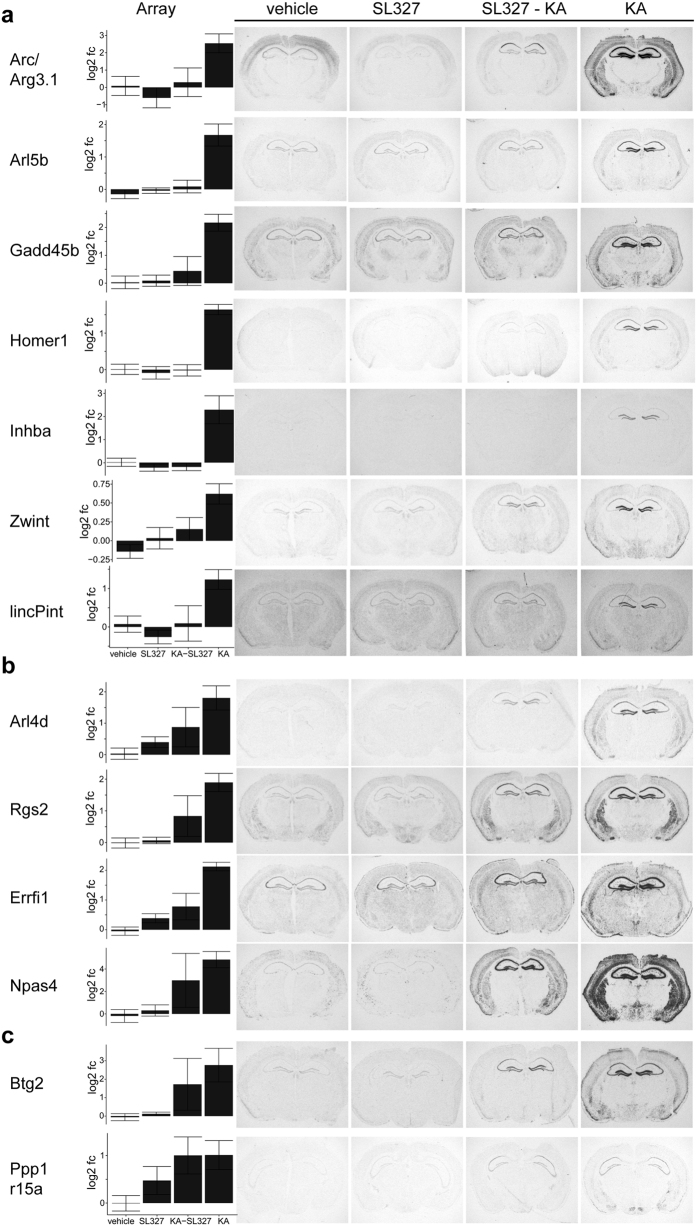
Expression of selected activity-regulated MEK dependent and independent genes. The bar diagrams (left) illustrate gene expression changes observed by microarray analyses. Autoradiograms of coronal sections of mice treated with vehicle, with the MEK inhibitor SL327, SL327 and kainic acid (KA), or KA and sacrificed 1 hour after seizure onset or after vehicle injections or 2 hours after SL327 injections. Radioactive *in situ* hybridizations of sections were conducted in parallel on one glass slide using gene specific antisense RNA probes. (**a**) Validation of MEK dependent genes, (**b**) partially MEK dependent genes, and (**c**) MEK independent genes are shown. CA1, CA1 area of the hippocampus; DG, dentate gyrus.

To validate our data, we analysed changes in gene expression by *in situ* hybridizations of brain sections of mice treated with vehicle, SL327, kainic acid, or SL327 combined with kainic acid. Each gene was analysed on sections of three different animals sacrificed 1 h after seizure onset or of time matched controls. The *in situ* hybridization analyses are, except for Btg2 see below, in good accordance with the results obtained from the microarrays and demonstrate that activation of Arc/Arg3.1, Arl5b, Gadd45b, Homer1, Inhba, and lincPint transcription in the hippocampus is hampered by MEK inhibition, whereas induction of Arl4d, Npas4, Rgs2, Errfi1 and Btg2 is only partially reduced by the inhibitor, and induction of Ppp1r15a is not effected by MEK inhibition (Fig. 5). *In situ* hybridizations revealed also the spatial induction pattern of these genes. For Arc/Arg3.1 and Gadd45b, we observed that seizures in the presence of the MEK inhibitor resulted in almost complete loss of induction with only modest signal detectable in the CA1 area and an almost complete inhibition in the dentate gyrus (Fig. 5a). The expression pattern of the partially MEK dependent genes visualized by *in situ* hybridization was identical after seizures in the presence or absence of the MEK inhibitor but expression levels were reduced throughout the hippocampus when the MEK inhibitor was applied (Fig. 5b). For Btg2 a strong interanimal variation in the microarray analysis was observed for animals treated with MEK inhibitor and kainic acid (Fig. 5c) and it was classified as MEK independent. Whereas the *in situ* hybridization analysis rather suggests that Btg2 is partially MEK dependent.

To further test the reliability of the microarray analysis we investigated the expression of Zwint. Its induction has a log fold change below 0.9. Therefore, it was excluded due to our criteria. Otherwise it would have been classified as a MEK dependent activity-regulated gene (Fig. 4a). *In situ* hybridizations corroborated the microarray results and Zwint expression was hardly detectable under control conditions, not induced by the inhibitor, but induced by seizures. The inhibitor prevented the induction although transcript levels were slightly higher than in controls (Fig. 5a). In conclusion, *in situ* hybridization analysis of 13 genes confirms the induction patterns observed in our microarray analysis.

### LincPint, a novel activity dependent long non-coding transcript

**Figure 6:**
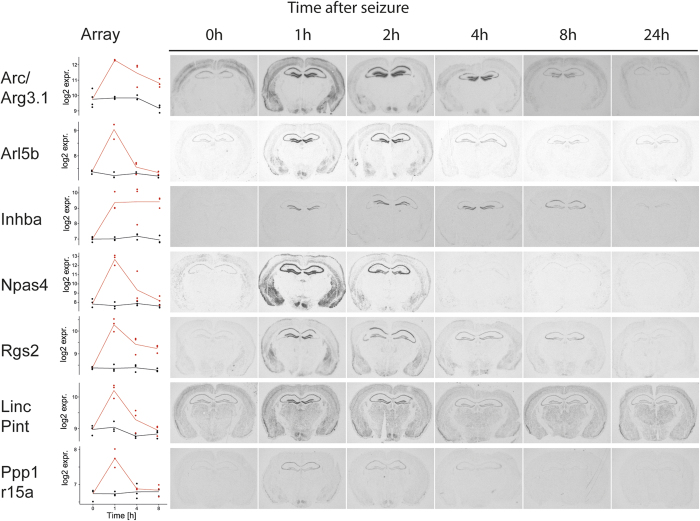
Examples of the temporal and spatial expression of activity-regulated genes at different time points after seizure onset. Plots of the time course and the ratio of induction based on the microarray analyses (left). The red lines represent expression kinetics at 0, 1, 4, 8 h after seizure onset, the black lines expression kinetics of vehicle treated time matched controls. Autoradiograms of coronal sections of mice sacrificed at indicated times after seizure onset. Radioactive *in situ* hybridizations of sections were conducted in parallel on one glass slide using gene specific antisense RNA probes.

We identified lincPint, a long intergenic non-coding RNA (lincRNA) as activity-regulated MEK dependent transcript, which has so far not been described as regulated by neuronal activity (Figs 4 and 5). To compare the expression of lincPint with known activity-regulated genes, we investigated its expression at different time points after seizure onset. Hippocampi of mice sacrificed 1, 4, or 8 hours after seizure onset were subjected to microarray analysis. In addition, animals were sacrificed at 1, 2, 4, 8, or 24 hours after seizure onset and brain sections were analysed by *in situ* hybridizations. LincPint is expressed on a low level in most regions of the brain and induced by seizures in the hippocampus with maximal induction after 1 hour (Fig. 6). LincPint transcripts are evenly induced in the hippocampus and downregulated to basal levels already 4 h after seizure onset. The induction kinetic was similar to the induction of Arl5b and Npas4. Other activity-regulated genes, such as Arc/Arg3.1, Inhba, and Rgs2 are induced for a longer time period. Inhba presents a complex hippocampal expression pattern that changes between the dentate gyrus and the CA1 area over time. The expression pattern of Rgs2 changes as well after induction. Expression is strongly induced 1 h after seizures in the dentate gyrus and in CA1, reduced already 2 h after seizures, evenly distributed in the hippocampus after 4 h, after 8 h strongly expressed in CA3, and reduced to baseline level after 24 h. We analysed as well the induction kinetic of the MEK independent gene Ppp1r15a and observed an induction in the hippocampus 1 and 2 hours after seizure onset followed by a rapid reduction of expression (Fig. 6).

### Alternatively spliced variants of the lncRNA Neat1 are differentially regulated by MAPK/ERK

**Figure 7:**
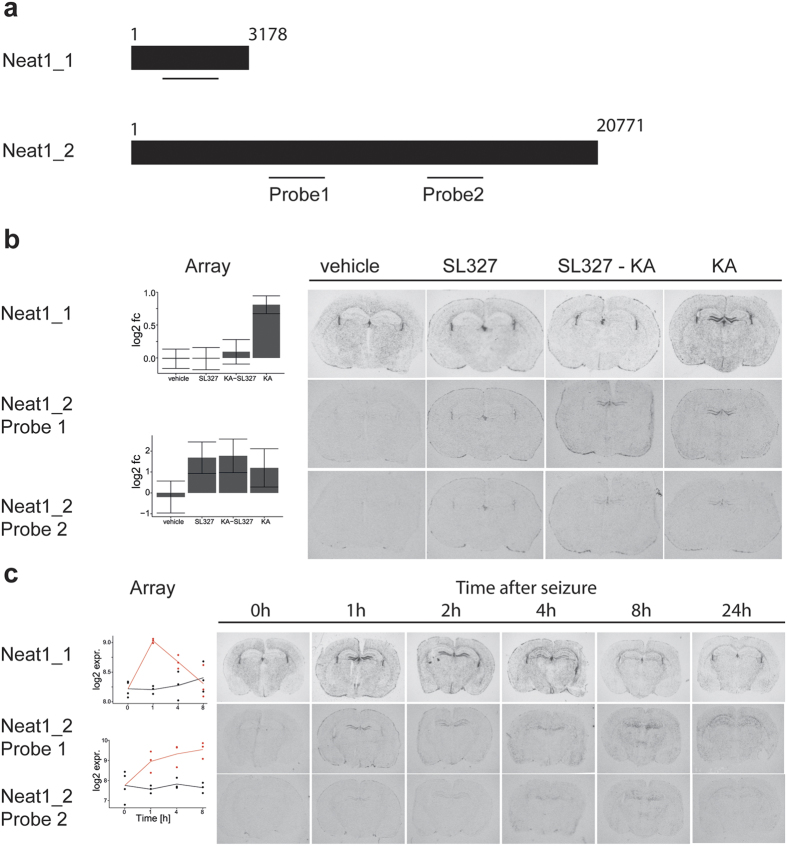
Activity dependent expression of Neat1 splice variants. (**a**) Neat1 is expressed as a short (Neat1_1 3178 bp) and a long (Neat1_2 20771 bp) splice variant. Black bars represent alternative Neat1 transcripts. Horizontal lines indicate position of specific *in situ* hybridization probes used for detection of Neat1. Two alternative probes were used to detect Neat1_2 transcripts. (**b**) Bar diagrams illustrate gene expression changes observed in the microarray analyses (left). Autoradiograms of coronal sections of mice treated with vehicle; SL327; SL327 and kainic acid (KA); or KA and sacrificed 1 hour after seizure onset, 1 hour after vehicle injections, or 2 hours after SL327 injection. Radioactive *in situ* hybridizations of sections were conducted in parallel on one glass slide using gene specific antisense RNA probes. (**c**) Plots of the time course and the ratio of induction based on the microarray analyses (left). The red lines represent expression kinetics at 0, 1, 4, 8 h after seizure onset, the black lines indicate expression kinetics of vehicle treated time matched controls. Autoradiograms of coronal sections of mice sacrificed at indicated times after seizure onset. Radioactive *in situ* hybridizations of sections were conducted in parallel on one glass slide using gene specific antisense RNA probes. The exposure time for all *in situ* hybridizations shown was 7 days for the Neat1_1 probe, and 60 days for the Neat1_2 probes.

We identified the lnc RNA Neat1 (nuclear enriched abundant transcript 1), also named MEN, as an activity-regulated transcript (Fig. 7). Neat1 is expressed as two splice variants differing in length. One, named Neat1_1 or MEN-β, has been previously described as an abundant 3.2 kb transcript and the other, named Neat1_2 or MEN-ε, as a less abundant 20 kb transcript^30^,^31^. Our microarray analysis suggested a very low expression level of Neat1_2 and in contrast a moderate and 3–4 times higher expression level of Neat1_1 in the murine hippocampus. In agreement, the short isoform was detected by radioactive *in situ* hybridization in the brain after 7 days of exposure, but the long isoform was undetectable at this exposure-time and became detectable after 30 days exposure. We observed an activity-dependent induction of both variants (Fig. 7b). Predominant induction in the dentate gyrus of the short variant, Neat1_1, lasted for 1 to 4 hours after seizure onset and was MEK dependent (Fig. 7b,c). In contrast, induction of the long variant, Neat1_2, lasted longer and was MEK independent (Fig. 7b,c). The spatial distribution of the Neat1_2 transcripts after induction was dynamic. Neat1_2 expression was restricted to the dentate gyrus 1 to 4 h after seizure. 8 and 24 h after seizure transcripts were more dispersed over the hippocampus (Fig. 7c). The expression of Neat1_2 after vehicle treatment was not detectable (Fig. 7b). Increased Neat1_2 expression after MEK inhibition, after seizures, and after combination of MEK inhibition and seizures was detected by microarray analysis. *In situ* hybridizations revealed a distinct and differential spatial induction in the fimbria of the hippocampus and paraventricular areas and only minor induction in the hippocampus after inhibitor application only. This expression pattern resembled the expression of Neat1_1 under control conditions (Fig. 7b). Seizures alone or in combination with the MEK inhibitor resulted in a distinct induction of Neat1_2 in the dentate gyrus. The observed activity-regulated expression of Neat1 variants and their differential regulation by the ERK signalling pathway links this pathway to the regulation of activity-dependent alternative splicing.

## Discussion

Sustained ERK activation plays a key role in synaptic plasticity in the mammalian hippocampus^6^,^9^,^10^. Synaptic stimulation leads to activation of MEK and results in ERK phosphorylation. ERK translocates from synaptic sites to the nucleus by interacting with protein messengers, such as Jacob, enters the nucleus and alters the transcriptional program of neurons^6^,^9^,^32^. So far, the identity of the specific genetic program targeted by ERK in the hippocampus has not been revealed.

The goal of the present study was to identify ERK dependent and independent activity regulated transcriptional signatures in the hippocampus *in vivo*. One strategy to induce activity regulated gene expression employs the generation of seizures to provoke robust, strong and synchronized induction of neuronal activity in the hippocampus of rodents. In fact, most of the transcripts that are known to be modulated after learning were initially identified using seizure protocols (reviewed in refs 33,34). We demonstrate ERK phosphorylation and its inhibition in the hippocampus after seizures. Phosphorylated ERK is predominantly found in granule cells in the dentate gyrus. In other parts of the hippocampus phosphorylated ERK was detected only in dendritic and axonal layers. The distinct pattern of ERK phosphorylation in the hippocampus in response to increased global neuronal activity provoked by seizures is in agreement with previous studies employing kainic acid or electroconvulsive seizures or long term potentiation (LTP)^24^,^27^,^35^. We used seizures combined with the pharmacological intervention of MEK activation as an *in vivo* model to determine genome wide the transcriptional program initiated by the MAPK/ERK signalling pathway after neuronal activity in the murine hippocampus. We blocked the MAPK/ERK signaling pathway by systemic administration of the potent MEK inhibitor SL327, a structural analog of the MEK inhibitor U0126. SL327 crosses the blood-brain barrier and has been favored for systemic administration^6^,^36^. SL327 has been characterized as a highly selective MEK inhibitor with no effect on a variety of other kinases, including PKA, PKC, or CamKII^15^,^28^. Our survey demonstrates that the induction of a large number of activity-regulated genes in the hippocampus is indeed dependent on ERK phosphorylation. This supports the concept that MAPK/ERK signalling is essential in activity dependent gene induction. Among the identified genes are well characterized immediate early genes crucially involved in plasticity related events, such as Arc/Arg3.1 and Homer1. Expression of both genes has been previously shown to depend on ERK activity^24^,^25^,^26^,^37^. However, the survey demonstrates that ERK activation is not sufficient for the induction of all activity regulated genes, because we identified a small number of partially MEK dependent genes. The neuronal MAPK/ERK signalling pathway targets different transcription factors which are in addition modulated by other signalling pathways acting in parallel. Taken this into account, activity regulated expression of partially MEK dependent genes might be conveyed by other parallel acting pathways, but these could be modulated through crosstalk with the MAPK/ERK pathway. Such a crosstalk might lead to the partial reduction of activity dependent expression when the MAPK/ERK pathway is inhibited. In addition, genes regulated by parallel pathways might be affected by MEK inhibition, as shared feedback regulation within MAPK/ERK signalling can lead to the activation of parallel signalling pathways^29^ and as a consequence to the induction of genes by MEK inhibition, as was observed for Dusp1, Col6a5 and Neat1_2.

We identified a small group of MEK independent genes that seem functionally unrelated. The detection of MEK independent genes demonstrates that the pharmacological intervention of ERK phosphorylation does not have pleiotropic effects on the general transcriptional machinery. We validated the activity regulated induction of the MEK independent gene Ppp1r15a (protein phosphatase 1 regulatory subunit 15a, which is also named growth-arrest- and DNA-damage-induced transcript 34, Gadd34). Ppp1r15a and protein phosphatase 1 (PP1) form a complex that dephosphorylates eukaryotic Initiation Factor 2α (eIF2α) which is regarded as a critical step in restoring general protein synthesis and promotes cell recovery from stress^38^. In addition, PP1 plays a role in modulating synaptic plasticity^39^ and negatively regulates ERK^40^, but so far Ppp1r15a function has not been addressed in this context.

Sustained synaptic stimulation leads to activation of ERK. Phosphorylated ERK enters the nucleus and alters the transcriptional program of neurons^3^,^4^. The downstream targets of the MAPK/ERK pathway modulating gene expression are directly or indirectly activated transcription factors. ERK directly activates the serum response element- (SRE-) binding protein ELK-1 whereas the cAMP response element-binding factor, CREB, is indirectly activated by intermediary kinases^6^,^12^. Several of the genes identified in this study, such as Btg2, Inhba, or Npas4, are reported CREB targets^41^ and belong to both identified groups of MEK dependent and partially MEK dependent genes. This corroborates the notion that CREB is one MAPK/ERK target. However, CREB can be also activated through other signaling pathways and conversely MAPK/ERK targets also other transcription factors. Accordingly, certain transcription factors can be modulated by alternative kinase signalling pathways to regulate broad and complex genetic programs.

The present survey analyzed hippocampal gene induction provoked by chemically induced seizures. Our validation experiments employing *in situ* hybridization of brain sections revealed induction of some transcripts in brain areas other than the hippocampus, e.g. Arc/Arg3.1, Rgs2, and Npas4 were as well induced in the cerebral cortex, the amygdala, and the striatum although to varying degrees. In these brain areas most but not all of the activity-regulated induction appeared MEK dependent suggesting brain region-specific modulation of MAPK/ERK signaling. A number of studies demonstrated a requirement for MAPK/ERK activity in several forms of plasticity in the hippocampus, but as well in the amygdala, the striatum and the cerebral cortex^6^,^42^. However, the precise modulators of the signaling pathway and the targets for MAPK/ERK in each of the different brain regions might differ.

ERK signalling has been shown to induce changes in the geometry of the nucleus in response to neuronal activity in hippocampal neurons^43^ and to regulate epigenetic changes by modulating histones^44^,^45^. Both mechanisms may underlie chromosomal decondensation which is required for transcriptional changes. Remodelling of chromosomal DNA depends on posttranslational modifications of histones, such as phosphorylation of histone H3 by mitogen and stress-activated kinase 1 (MSK1) which is activated by MAPK/ERK signalling^44^,^45^. The role of MAPK/ERK in histone H3 phosphorylation has been extensively investigated. Moreover, there is accumulating evidence that phosphatases such as PP1 are also important determinants in this process^44^,^45^.

In addition to protein coding transcripts, we identified activity-regulated long non-coding (lnc) RNAs. Dynamic expression profiles of several lncRNA after hippocampal LTP induction have been demonstrated recently^46^. LncRNAs have been suggested as nuclear key factors that organize nuclear sub-structures, modulate chromatin state, and regulate gene expression^47^. We demonstrate MEK dependent activity-regulated expression of the lncRNA lincPint and visualize its inducible expression pattern in the brain. LincPint is ubiquitously expressed and knockout mice are reduced in size and body weight^48^. LincPint localizes to the nucleus and interacts with the Polycomb Repressive Complex 2 (PRC2). PRC2 represses gene expression by catalysing methylation of histones and modulating chromatin structure. LincPint alters PRC2 binding to specific genomic loci^49^. Expression of anti-sense oligos against lincPint in MEF cells resulted in upregulation of the activity regulated genes Arc/Arg3.1, Gadd45b, and Egr2^49^. These findings suggest lincPint as a candidate for conveying epigenetic changes initiated by MAPK/ERK. These might comprise suppression of specific gene transcription to downregulate genes following their induction together with lincPint.

In addition, we identified Neat1 as an activity dependent lncRNA and find that splice variants of Neat1 are differentially regulated by MAPK/ERK. It has been shown previously that Neat1 is ubiquitously expressed^50^ and transcripts are increased in human epileptic brain and in depolarized human neuroblastoma cells^51^. Neat1 localizes exclusively to paraspeckles and serves as an architectural component of these nuclear bodies^30^,^50^. The cell nucleus contains distinct classes of subnuclear bodies, inlcuding nucleoli, paraspeckles, splicing speckles, Cajal bodies, and PML bodies^52^. The latter have been shown to be dynamically modulated in number and size by seizures and PML bodies have been suggested to be associated with activity-dependent nuclear alterations^53^. A proposed function of paraspeckles is subnuclear sequestration of nuclear proteins, the regulation of expression of adenosine-to-inosine hyper-edited mRNAs and nuclear retention of target transcripts^54^,^55^. The knockdown of Neat1 leads to the disintegration of paraspeckles^30^,^50^. Two Neat1 splice variants have been described Neat1_1, a short 3 kb polyadenylated transcript, and Neat1_2, a long 20 kb non-polyadenylated transcript^31^. Neat1_2 constitutes paraspeckles whereas Neat1_1 cannot induce nuclear body formation by itself^31^,^50^, although expression of Neat1_1 seems to increase the number of paraspeckles^30^. In agreement with previous analysis^56^, we observed a low expression of Neat1_1 in the brain where expression of Neat1_2 was barely detectable. We demonstrate the MEK dependent induction of Neat1_1 in the dentate gyrus after seizures. Neat1_2 expression is undetectable under control conditions and can be induced already by MEK inhibition alone. The pattern of expression resembled that of the shorter form under control conditions. The induction of Neat1_2 by neuronal activity is independent of MEK and lasts longer than the induction of Neat1_1. These findings indicate that MAPK/ERK signalling suppresses expression of Neat1_2 under control conditions, but nevertheless can be induced by synaptic activity. In contrast, under control conditions the basal expression of the shorter splice variant is MAPK/ERK independent but induction following neuronal activity is strictly dependent on MAPK/ERK activation. This substantiates that MAPK/ERK signalling conveys activity dependent differential splicing of Neat1. This in turn might underlie activity dependent nuclear transformations.

## Materials and Methods

### Tissue preparation

Animal husbandry and experiments were authorized under German regulations on animal welfare in accordance with the European Communities Council Directive of 22 September 2010 (2010/63/EEC) and approved by local authorities of the city-state Hamburg (Freie und Hansestadt Hamburg, Behörde für Gesundheit und Verbraucherschutz, Fachbereich Veterinärwesen, No 65/14). 3 months old male C57/bl6 mice were housed with a 12 hours dark-light-schedule and experiments were performed during the dark cycle always at identical time points. Kainic acid (Ascent scientific) (20 mg/kg, dissolved in PBS), SL327 (Sigma) (50 mg/kg, dissolved in dimethylsulfoxide (DMSO) and mixed 1:1 with PBS) or similar amounts of DMSO/PBS were administered by intraperitoneal injection. Mice treated with kainic acid and SL327 were administered with SL327 1 hour before kainic acid injections. Animals were sacrificed at given time points after onset of seizure or at corresponding time points after SL327 or vehicle injections as described before^57^.

### Immunohistochemistry

Mice were anesthetized by intraperitoneal injection of urethane (1.6 g/kg bodyweight), transcardially perfused with 4% paraformaldehyde in phosphate buffered saline (PBS, pH 7.4) and brains were dissected and postfixed overnight in 4% paraformaldehyd. 40μm thick sections were cut using a vibratom (Leica) and incubated with pERK antibody (Cell Signalling #9101), dilution 1:1000; Arc/Arg3.1 antibody^58^, dilution 1:1500 or p-CaMKII (Thr286)(D21E4) (Cell Signalling #12716), dilution 1:3000. Immunoreactivity was detected using the avidin-biotin system (Vectastain, Vector Labs). Finally, sections were developed using diaminobenzidin (Sigma) as a chromogen. All experiments were performed with four replicate animals.

### Microarray hybridization

For RNA isolation hippocampi were dissected from fresh brains, flash frozen and stored at −80 °C. Total RNA was isolated using TRItidy-reagent (Applichem), followed by an additional purification step using RNEasy columns (Qiagen), quantified by UV-spectroscopy and its quality verified using a LabChip BioAnalyzer (AGILENT Technologies). The amplification and labeling of RNA samples were conducted according to the manufacturer’s instructions (Affymetrix). One μg from each sample was transcribed to cDNA using an oligo(dT)24 primer containing a T7 RNA polymerase promoter. After RNAse H-mediated second strand cDNA synthesis, the product was purified and served as a template in the subsequent *in vitro* transcription reaction. Biotin-labelled cRNA was prepared from double-stranded cDNA by *in vitro* transcription using the GeneChip RNA transcript labelling kit (Affymetrix). After clean-up, biotin-labelled cRNA was fragmented by alkaline treatment [40 mmol/l Tris-acetate (pH 8.2), 100 mmol/l potassium acetate, and 50 mmol/l magnesium acetate] at 94 °C for 35 minutes. 15 μg of each cRNA sample was hybridized for 16 h at 45 °C to an Affymetrix Mouse Exon Array 1.0 ST GeneChip covering the complete transcribed mouse genome. Chips were washed and stained with streptavidin-phycoerythrin using a fluidics station according to the protocols recommended by the manufacturer. Finally, arrays were scanned at 1.56-μm resolution using the Affymetrix GeneChip System confocal scanner 3000.

### Data analysis

Data from GeneChip microarrays has been deposited in the NCBI Gene Expression Omnibus (GEO) and is accessible through the GEO Series accession number GSE88723 (available prior to publication under: https://www.ncbi.nlm.nih.gov/geo/query/acc.cgi?token=wtgfgkwczzgtfob&acc=GSE88723). Expression analysis was performed in R (http://www.bioconductor.org), using the bioconductor packages “oligo” for normalisation and “limma” for differential expression calling. Briefly, raw CEL files were red in, normalised using robust multi-array average (rma) of the package oligo with the parameter target = ”full”. After annotation of the probe sets, probe sets without annotation were removed, as were probe sets of category other than “main”. Next, only probe sets that had normalized expression values above the median in at least three arrays were retained for further analysis. Differential expression calling was done using the Linear Models for Microarray Data” (limma) package by first fitting a linear model using limma’s lmFit to all condition, and subsequently fitting contrasts between the indicated groups. Differential expression was called using the decide Test function with Benjamini-Hochberg multiple testing correction first applied on kainic acid vs. vehicle control to select all induced genes. Subsequent testing was limited to the set of differential genes (kainic acid vs. vehicle) and multiple testing was corrected for separately using Benjamini-Hochberg. MEK inhibitor sensitive genes were selected without multiple-testing correction, to limit false negatives. Error was estimated using eBayes method and thresholds of FDR < 0.05 or p < 0.05 were applied.

### *In situ* hybridization

For *in situ* hybridizations, animals were sacrificed by cervical luxation at indicated time points after onset of the first seizure (each time point, n = 3), control animals were sacrificed 30 minutes after vehicle injection (n = 3).

Total brains were flash frozen using liquid nitrogen and stored at −80 °C until cryosectioning. *In situ* hybridization was essentially performed as described before^59^. In brief, antisense RNA probes labelled with [α-^35^S]-UTP were generated according to the manufacturer’s instructions (Promega). 20 μm cryosections of brains were fixed in 4% paraformaldehyde-PBS, acetylated, dehydrated and hybridized at 55 °C for 18 h. Ribonuclease A treatment was performed for 30 min at 37 °C. Following a high stringency wash in 0.1x saline sodium citrate buffer at 55 °C, slides were exposed to X-ray films (Kodak Biomax MR; Amersham Bioscience) for 72 h. Specificity of signals was verified by comparing antisense to sense controls. DNA templates were generated by PCR or restriction digest from full-length cDNA clones and cloned into pBSK (Stratagene). For a complete list of templates see Table 1. Each gene was analysed at least on sections of three different animals of one experimental group.

## Additional Information

**How to cite this article:** Blüthgen, N. *et al*. Profiling the MAPK/ERK dependent and independent activity regulated transcriptional programs in the murine hippocampus *in vivo. Sci. Rep.* **7**, 45101; doi: 10.1038/srep45101 (2017).

**Publisher's note:** Springer Nature remains neutral with regard to jurisdictional claims in published maps and institutional affiliations.

## Supplementary Material

Supplementary Information

## Figures and Tables

**Table 1:** Genes validated by *in situ* hybridizations, respective nucleotides of templates and accession numbers.

Gene	Nucleotides	GeneBank^TM^ Accession no.
Arc/Arg3.1	589–1198	NM_018790
Arl4d	466–1306	NM_025404
Arl5b	760–2400	NM_029466
Btg2	53–529	BC138639.1
Errfi1	255–2317	NM_133753
Gadd45b	226–708	NM_008655.1
Homer1a	1191–2136	NM_011982
Inhba	1053–1530	NM_008380
Npas4	885–1581	BC129861.1
Neat1_1	1067–1789	NR_131212.1
Neat1_2 (1)	3203–3826	NR_131212.1
Neat1_2 (2)	14132–14791	NR_131212.1
Pint	1–402	NR_110469.1
Ppp1r15a	395–846	NM_008654.2
Rgs2	32–667	NM_009061.4
Zwint (var.1)	815–1516	BC034870
