# Supplementary material for: Profiling the MAPK/ERK dependent and independent activity regulated transcriptional programs in the murine hippocampus in vivo
Source: Sci Rep. 2017 Mar 28;7:45101. doi: 10.1038/srep45101 (PMC5368636; doi:10.1038/srep45101)
Supplement: Supplementary Information [file srep45101-s1.pdf]

Profiling the MAPK/ERK dependent and independent activity regulated transcriptional programs in the murine hippocampus in vivo

Nils Blüthgen, Mirjam van Bentum, Barbara Merz, Dietmar Kuhl, Guido Hermey

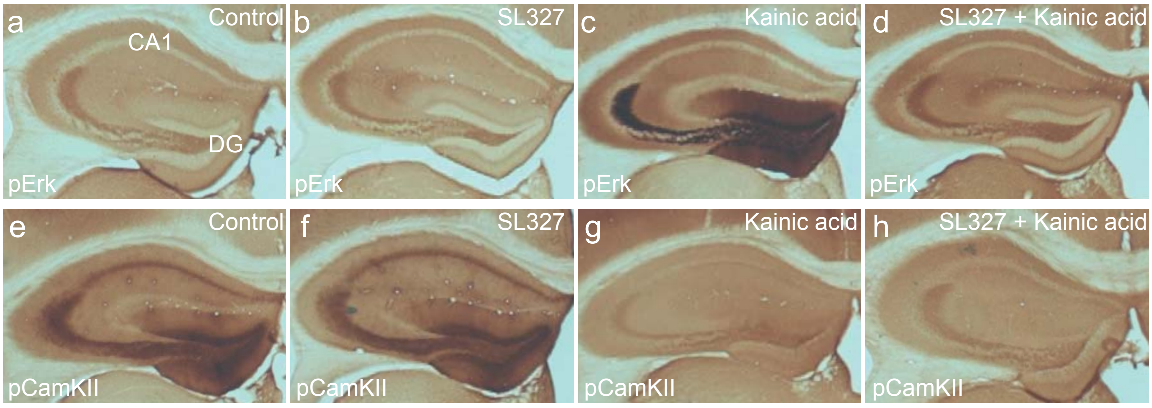

Supplemental Figure 1. The MEK inhibitor SL327 blocks seizure induced ERK phosphorylation but has no effect on autophosphorylation of CaMKII.
The inhibitory domain of the Calcium/calmodulin-dependent protein kinase II (CaMKII) is within the same polypeptide. Autophosphorylation of Thr286 occurs as an inter-subunit reaction and modulates CaMKII kinase activity[1]. Previous studies demonstrated that seizures cause a dramatic loss of CamKII Thr286 phosphorylation[2]. Parallel coronal mouse brain sections were immunostained for p-ERK (a-d) or for p-CaMKII (Thr286) (e-h). Mice were treated with vehicle (a, e); with SL327 for 90 minutes (b, f); treated with kainic acid and sacrificed 15 minutes after onset of seizures (c, g); or treated with SL327 60 minutes before intraperitoneal kainic acid injections and sacrificed 15 minutes after onset of seizures. The monoclonal p-CaMKII antibody recognizes endogenous CaMKII-α only when phosphorylated at Thr286 and CamKII-β, and CamKII-γ only when phosphorylated at Thr287. Note that SL327 treatment does not alter the level of CaMKII autophosphorylation at Thr287. CA1, field CA1 of the hippocampus; DG, dentate gyrus.

Supplemental references

1. Lisman, J., Schulman, H. & Cline, H. The molecular basis of CaMKII function in synaptic and behavioural memory. Nat Rev Neuroscience 3, 175-190, doi:10.1038/nrn753 (2002)

2. Dong, Y. & Rosenberg, H.C. Brief seizure activity alters Ca2+/calmodulin dependent protein kinase II dephosphorylation and subcellular distribution in rat brain for several hours. Neurosci Lett 357, 95-98, doi:10.1016/j.neulet.2003.11.069 (2004)
